# Loco-Regional and Systemic Chemotherapies for Hepato-Pancreatic Tumors: Integrated Treatments

**DOI:** 10.3390/cancers12102737

**Published:** 2020-09-24

**Authors:** Girolamo Ranieri, Carmelo Laface

**Affiliations:** Interventional Oncology Unit with Integrated Section of Translational Medical Oncology, National Cancer Research Centre, Istituto Tumori “Giovanni Paolo II”, Viale Orazio Flacco 65, 70124 Bari, Italy; carmelo.laface@gmail.com

**Keywords:** primary and metastatic liver tumors, pancreatic cancer, hepatic arterial infusion, transarterial chemoembolization, loco-regional therapies

## 1. Liver: Site of Primary Tumors and Metastases

This Special Issue of *Cancers*, titled “Loco-Regional Arterial Chemotherapies Alone or in Combination with Systemic Treatments for Primary and Secondary Hepato-Pancreatic Tumors”, focuses on new possible strategies to treat only liver disease (or mainly liver disease) through the combination of loco-regional and systemic chemotherapies.

This aim was borne because many types of tumors grow or metastasize, mainly in the liver, leading to its functional failure and patient death despite other organs usually having a lower burden of disease.

Hepatocellular carcinoma (HCC) is the most common primary hepatic malignancy and is the fourth leading cause of cancer-related deaths worldwide. Systemic therapy is available nowadays, but treatments for advanced HCC offers the benefit of only a few months of survival [1].

Intrahepatic cholangiocarcinoma (ICC) is the second most common primary hepatic malignancy. It has a poor prognosis, with a median survival of 3–8 months without treatment. At the same time, the gold standard chemotherapy for advanced ICC, that is, the combination of gemcitabine with cisplatin, confers only a modest benefit in terms of overall survival (OS) [2].

Colorectal cancer (CRC) is the fourth most frequently diagnosed cancer and the second leading cause of cancer-related death in the United States. CRC is the second leading cause of cancer in both sexes in Italy, with a 5-year survival rate of 65%. Approximately 50% to 60% of patients affected by CRC develop metastases and the liver represents the most frequent metastatic site, with almost 90% of cases leading to death in most patients due to its functional failure [3].

Pancreatic cancer is the seventh most common cancer in the European Union. Despite the fact that it is an infrequent cancer, it corresponds to the fourth most common causes of cancer mortality [4]. Advanced cancer is characterized by a very poor prognosis, with just 11.6 months of OS afforded by the best treatment available to date, the chemotherapy regimen consisting of oxaliplatin, irinotecan, fluorouracil, and leucovorin (FOLFIRINOX) [5]. Pancreatic cancer metastasizes almost exclusively to the liver. 

## 2. The Rational of Integrated Therapeutic Strategies 

The poor prognosis of the aforementioned tumors and the main arterial perfusion through the hepatic artery of liver malignancies lead to the use of selective intraarterial therapies, whether or not these are combined with systemic chemotherapy.

Therefore, these tumors could be better treated by means of targeted locoregional therapies such as conventional transarterial chemoembolization (cTACE), drug-eluting bead TACE (DEB-TACE), hepato-pancreatic artery infusion chemotherapy (H-PAI) and hepatic arterial infusion chemotherapy (HAI).

cTACE is based on the intra-arterial injection of an emulsion of lipiodol and a chemotherapeutic such as doxorubicin, epirubicin, mitomycin, cisplatin or irinotecan, followed by the selective occlusion of the tumor arterial blood supply through embolic agents [6,7]. This approach allows for ischemic tumor necrosis and prevent rapid washout of the chemotherapeutic drug [8,9,10]. However, postembolization hypoxia could act as angiogenesis stimulator. In fact, oxygen deprivation leads to the expression of Vascular Endothelial Growth Factor (VEGF), the main pro-angiogenic factor, by the microenvironment cells, as demonstrated in human and animal tumor models [11]. VEGF level has been associated with tumor angiogenesis, growth, relapse and metastasis. For this reason, many studies about HCC suggest the association of TACE with anti-angiogenic drugs such as sorafenib and regorafenib, suppressing vessel growth [12].

Instead, DEB-TACE provides for the use of microspheres that sequester, by means of exploiting ionic bonds, and then slowly release, with controlled pharmacokinetics, the chemotherapeutic agent inside the target lesion [13]. 

HAI and H-PAI consist of perfusion with chemotherapeutics, through a catheter or pump, of the target organ. These approaches allow for selective chemotherapeutic delivery to the most involved organ by the tumor. Moreover, locoregional therapies combine the advantages of an increased pharmacological effect in the target organ with a reduction in systemic side effects. For this reason, drugs with large first-pass extraction, high total body clearance and a short plasma half-life are usually used, such as oxaliplatin, 5-fluorouracil, gemcitabine and irinotecan. However, first-pass extraction not only limits toxicity, but also the systemic effects of chemotherapy, so locoregional therapies are often combined with systemic therapy to maintain disease control [7,14,15,16,17,18]. 

## 3. Future Directions

In the literature, interesting data can be found regarding the clinical results derived from the abovementioned locoregional therapies for the management of primary and secondary liver malignancies in selected patients. Nowadays, these approaches are performed only by a few centers in the world, but many more patients could be treated with these techniques, improving the survival benefit. Therefore, this Special Issue aims to increase the awareness about the benefits of combined treatments by discussing the various clinical results that can be obtained. 
